# Building the Generalist Physician to Support Adolescence and Emerging Adulthood: A Narrative Review

**DOI:** 10.7759/cureus.22533

**Published:** 2022-02-23

**Authors:** David Chartash, Laura Hart

**Affiliations:** 1 School of Medicine, University College Dublin - National University of Ireland, Dublin, IRL; 2 Center for Medical Informatics, Yale School of Medicine, New Haven, USA; 3 Internal Medicine/Pediatrics, Nationwide Children’s Hospital, Columbus, USA

**Keywords:** undergraduate and graduate medical education, clinical clerkship, medicine-pediatrics, internal medicine & pediatrics, entrustable pofessional activities

## Abstract

Undergraduate medical education serves as a foundation for the medical student to develop the skills of a generalist physician. Given the "blurring" of the demarcations between childhood and adulthood and the increased scope of pediatric practice, an extra layer has been added to medical education which seeks to address care across the lifespan. While approaches have been developed to teach this layer, clerkship reform has not focused on advancing the clinical science of adolescence. Furthermore, as we look towards the vanguard of entrustable professional activities (EPA), specific attention to transition care for the adolescent has seen minimal attention. Drawing on prior examples of curriculum integration between specialties as well as solutions to complex care management from clinical reasoning, we suggest that attention to the development of the generalist physician requires attention to the combined medicine-pediatrics specialty.

## Introduction and background

Undergraduate medical education seeks to build a generalist frame for medical students as they learn from a wide breadth of clinical skills and experiences. In addition to explicit expectations from regulatory and accreditation bodies that students learn from a range of disciplines (such as medicine, pediatrics, surgery, obstetrics, and psychiatry), students are expected to learn in a variety of care settings (hospital and ambulatory) and care contexts (acute, chronic, continuing, preventive, and rehabilitative). As students move through these experiences, they acquire clinical skills particular to each of these facets of medicine and are assessed on their mastery of them [1]. Medical students are also responsible for applying these skills across medical disciplines into a practice that is capable of caring for any patient [2]. Currently, there is an explicit separation of pediatric and adult medicine in the formal curriculum. However, students must link the two as part of building their generalist frame in order to take care of both adults and children. In reality, the line between "pediatric" and "adult" has blurred over time, making the link between pediatric and adult all the more critical for students to consider [3,4]. Given these areas of overlap, more directed training around linking across the pediatric/adult divide may be needed in undergraduate medical education.

A number of historical developments and policy changes have resulted in the "graying" of the relatively black-and-white demarcations between pediatric and adult health care. Most notably, survival into adulthood has gone from a rare event to an expected outcome for numerous childhood-onset diseases, like Down syndrome [5], severe congenital heart defects [6], childhood cancers [7], and cystic fibrosis [8], to name a few. Our understanding of autism has grown substantially, and with it, the recognition that autism is much more common than previously thought. If we extrapolate from CDC data, approximately 1% of 20-somethings have autism [9]. Conversely, the incidence of historically "adult" diseases like type II diabetes continues to rise among adolescents [10]. In addition, the Affordable Care Act allows young people to stay on their parents' insurance until age 26, which may encourage young adults to delay seeking health care in adult settings or thinking about how they will manage their health independently [11]. In an effort to support young adults who are facing many life changes at once and in recognition of the significant brain development that occurs in the early 20s, the American Academy of Pediatrics has softened regarding a strict age limit for receiving care from pediatric providers and encourages patients and providers to talk about the appropriate timing for transfer to adult care [3].

These changes have added an extra layer to medical education, particularly for those training in primary-care-focused residency programs. For pediatric residents, it means building an understanding of young adulthood. For medicine residents, it means building an understanding of adolescence. For those of us in med-peds and, similarly, for our family medicine colleagues who also consider care across the lifespan, it means giving more explicit attention to the continuum. By extension, undergraduate medical students, who must also consider care across the lifespan, are now tasked with considering the overlap between pediatric and adult health care in a new way. In order to ensure medical students graduate with the requisite knowledge regarding this integration, more structured training integrating across the age continuum is needed.

## Review

Collaborative approaches to creating a generalist physician treating both adults and children (including the formal discipline of adolescent medicine) have been limited by the insufficient pedagogical emphasis in undergraduate medical education [12,13]. Most recently, success has been had through the development of longitudinal clinical clerkships, which emphasize an understanding of the continuum of care across undergraduate medical education. Longitudinal clerkships, however, do not provide the same experience as traditional block rotations with regards to specific pathological topic depth and are still dominated by disciplinary divides depending on teaching site location, limitingong the trajectory of learning, which culminates in the medical studen their ability for students to consider the pediatric/adult overlap [14]. Such endeavors serve to provide the knowledge and skills necessary for students to obtain transdisciplinary skills across the core clinical clerkships (including internal medicine and pediatrics) [15], reduce the fragmentation of the clerkship experience [16], and sequence the development of students to ensure that they obtain fundamental general clinical skills, such as presentation [17]. This model of longitudinality in the undergraduate curriculum has moved beyond clinical skills to encompass such topics as evidence-based medicine [18,19], clinical informatics [20], and social medicine [21]. In line with the aforementioned longitudinality, rather than clerkship reform seeking to add prototype information to the student’s medical knowledge that is insufficient to build an understanding of clinical science [22], clerkship reform has primarily focused on situating the student within the continuum of care to learn from experience as a means to support the development of skills in clinical science [23].

Examples from the graduate medical education experience provide examples to consider as we ponder ways to support medical students in gaining the skills and knowledge necessary to participate in the learning and practice of clinical science. One institution created a common curriculum for central line training. During intern orientation, all incoming residents who would be inserting central lines completed this training, creating a common institutional understanding of the procedure [24]. Education efforts aimed at students could similarly create common training around topics that are potentially addressed in a number of medical disciplines, such as contraception. Formal transition clinic experiences for residents have been proposed [25]. Initial data suggest that structured rotations where a pediatric and medicine resident see the same adolescent/young adult patient together may be an option for improving the skills of both pediatric and internal medicine residents in caring for this population. Residents who participated in these experiences report that they gained insights that improved their comfort in caring for this population, whether pediatric or internal medicine [26]. Case studies of adolescent/young adult health challenges with input from pediatric, internal medicine, and family medicine providers could provide parallel cross-disciplinary perspectives in undergraduate medical education.

Simply incorporating these kinds of educational opportunities into the undergraduate curriculum will not ensure that students can link pediatric and adult care. Students will need to be assessed on the skills as well. Entrustable Professional Activities (EPAs) are a means to understand how well a student performs the clinical skills necessary to perform as a physician [27]. Adequate performance on EPAs is necessary for students to move along the path toward autonomy and independent practice and requires a level of trust in the capacity of the physician as a clinical scientist. The skills related to history-taking and clinical documentation are particularly relevant with respect to a student’s ability to consider the continuum from pediatric to adult care. History-taking takes a different form for children than for adults, with content, participants, and focus altered [28]. Part of considering the "gray" area between pediatric and adult care for the student is recognizing when it might be beneficial to bring history-taking and note-writing skills from pediatrics into an adult setting and vice versa. As a result, the EPAs related to clinical documentation serve as an ideal place to assess students on these linking skills.

In the set of EPAs devised by the Association of American Medical Colleges [29], three EPAs relate directly to clinical documentation: EPA I (gather a history and perform a physical examination); EPA V (document a clinical encounter in the patient record); and EPA VII (form clinical questions and retrieve evidence to advance patient care). In addition to the EPAs associated directly with clinical documentation, EPA VIII (Give or receive a patient handover to transition care responsibility) is purposely described as a task of communication, separated into the functions of the transmitter and the receiver of information. Key aspects of this include an understanding of situational awareness and contingency planning, as well as ensuring closed-loop communication such that the transition of responsibility has occurred from the entrustable physician to those taking over. While the transition between pediatric and adult care is explicitly mentioned in the potential settings [30,31], it is not the scenario envisioned by the exemplars provided (which describe within-care setting shift-to-shift transition). Furthermore, the lack of confidence of residents in such a handover [32] suggests that the lessons of EPA VIII are not adequately being addressed in undergraduate medical education. As the pediatric to adult medicine transition is explicitly mentioned in EPA educational material, more emphasis on this context of adolescence would support a more nuanced understanding of EPA VIII itself.

The pediatric encounter requires a different script than the adult encounter, particularly including the parent as a decision-maker and examining the child’s unique social and medical context. Beyond the differences expected in such clinical skills between pediatric and adult encounters, which contribute to the task of documentation, models of clinical communication, such as the Calgary-Cambridge model [33,34], promote a generative model for medical students to gain proficiency in the skill of history-taking. Building towards autonomy, such a generative model requires that the student understand and develop an independent capacity for history-taking given a breadth of experience with patients and formal education [35,36]. An autonomous endpoint, therefore, expects an ideal reconciliation of the disciplines, care settings, and clinical contexts of medicine, developing a general set of clinical skills irrespective of the vocational training to be pursued in residency.

Along the trajectory of learning, which culminates in the medical student gaining a grounding in generalist medicine, the medical student’s reconciliation of clinical skills between pediatric and adult medicine takes into account the necessary differences in the clinical encounter and note-writing [37]. Taking into account the differences in the clinical encounter and documentation, disciplines such as internal medicine, pediatrics, family medicine, and obstetrics have different approaches to managing the complex environment of clinical care. For example, we have family medicine’s scope of practice and systems-based approach [38-40] or pediatric family-centered rounds [41], both of which aim for a more holistic approach to care beyond the patient, despite underlying practice demographics [42]. Fundamentally, the integration of disciplines and understanding of contexts shapes care, and with the med-peds physician caring for adolescence, we see a physician capable of operating within the complex intersection of multiple disciplines [43]. This is further evinced by the combination of pediatric, family medicine, and obstetric residents requiring further attention to skills, experience, and training in order to adequately deal with adolescence despite working within their individual scope of practice [44]. In seeking to educate the medical student and broaden their understanding of the skills of the generalist physician, more formal meta-cognitive strategies offer opportunities to learn from the disciplines surrounding adolescence. Students need the skills to think about how they think through a case just as much as they need the skills to think through the case itself. This includes awareness of and strategies to mitigate bias and recognition of an individual’s tendencies with respect to cognitive errors. These skills are key to meeting the goals of entrustable professional activities within the scope of generalist practice, which will be necessary for the 48% of physicians (for the 2021 NRMP match [45]) who matriculate into internal medicine, pediatrics, or family medicine programs. Furthermore, an understanding of generalist practice is valuable for any specialist who cares for both children and adults as they seek to make context-specific decisions on all levels of problems faced by patients. 

Meta-cognitive skills are but one component of the complex skill of clinical reasoning [46]. Students need education regarding the management of complexity, both medical and social, in order to support their cognitive ability to care for complex patients in practice [47,48]. Education strategies that bring together previously taught skills, such as reflection on previously completed clinical documentation, allow students to build an understanding of medical disciplines while also seeing examples of important meta-cognitive strategies. These integrated education strategies also support a larger goal of medical education: graduating students are more capable of dealing with the risk, uncertainty, and complexity inherent in modern medicine [49-51]. A clinical example of the value of the generalist skillset and meta-cognition purposed for clinical science is seen in the case of juvenile idiopathic arthritis (JIA) [52]. JIA is a complex disorder that can have extra-articular manifestations and can require extensive monitoring for complications of treatment. Moreover, the manifestations of the illness can change with age, with some people essentially no longer having the disease and others continuing to have life-long pain and physical limitations. A well-trained generalist would approach a new young adult patient with JIA by having some basic understanding of the disease as well as an openness to recognizing that this understanding may only partially fit with the patient’s experience of the illness. With that mindset, the generalist can have a productive visit with the patient and an appropriate care plan can be made. Empirically, the value of a generalist’s perspective from both medicine and pediatrics is seen in the management of epidemiologic [53] and clinical [54] compilations of the differences between child and adult, the pediatric and adult rheumatologist, and the changing manifestation of the disease.

## Conclusions

Given the goal of producing a physician capable of supporting children, adolescents, and adults through undergraduate medical education, an ideal generalist frame to manage an uncertain and complex clinical science is that which is bounded by the integration of medicine and pediatrics. This is not a task that anyone with a specialty can manage alone. For example, despite the changing demographic of patients seen by family medicine, their scope of practice and approach to systems-level thinking and care across the life course offers an approach different than the one seen by pediatrics or internal medicine. Undergraduate medical education should provide opportunities for students to see this wealth of perspectives, ideally in a way that provides opportunities for students to see how different specialties approach the same patient scenario. The specialty of medicine-pediatrics is uniquely positioned to lead this challenge alongside other specialties, as it stands not only at the point of adolescence but is most familiar with the integration of specialties to form a subject greater than the whole.
